# Removal of Lead from Water Using Calcium Alginate Beads Doped with Hydrazine Sulphate-Activated Red Mud as Adsorbent

**DOI:** 10.1155/2017/4650594

**Published:** 2017-12-31

**Authors:** A. Naga Babu, G. V. Krishna Mohan, K. Kalpana, K. Ravindhranath

**Affiliations:** Department of Chemistry, K L University, Green Fields, Vaddeswaram, Guntur, 522502, India

## Abstract

Calcium alginate beads doped with hydrazine sulphate-treated red mud are investigated as adsorbent for extracting lead ions from water using batch methods of extraction. Different extraction conditions are optimised for maximum lead extraction. Substantial amount of lead is removed, and the adsorption ability is found to be 138.6 mg/g. Surface characterization using FTIR, EDX, and FESEM confirms that lead is “onto” the surface of the adsorbent. Thermodynamic parameters, adsorption isotherms, and kinetics of adsorption are analysed. Adsorption is “physisorption” in nature and spontaneous. The adsorbent developed can be regenerated using 0.1 M HCl. Thus regenerated adsorbent can be used as the adsorbent for further removal of lead at least 10 times, and this enables the complete removal of lead from water by repetitive use of the regenerated adsorbent. The beads facilitate the easy filtration. The methodology developed is successfully applied for removing lead from industrial waste waters.

## 1. Introduction

The consumption of water containing lead ions is hazardous, and it is reported that it causes various ailments such as kidney and neurological problems, anaemia, brain hemorrhage, and even death [1, 2]. The maximum permissible limit is 10 ppb (WHO), and presently, zero lead concentration of waters is preferred [3]. Further, the lead is not biodegradable, and hence, the problems are amplified.

Intensive investigations are being carried out throughout the globe to remove lead from the waters and they have one or other disadvantage, and a globally acceptable eco-friendly, economical, and effective method is still eluding the researcher. In this contest, the unconventional methods are attracting the researchers. Our research group has developed some methodologies based on adsorbents derived from biomaterials for removing various polluting ions such as chromium (VI) [4, 5], zinc [6], aluminium (III) [7], fluoride [8, 9], nitrite [10], ammonia [11], phosphate [12], and dyes [13] from water.

Red mud is a waste product from aluminium industries, and its adsorption nature towards various pollutants is being investigated [14–18]. The uses of treated red mud in the removal of lead are reported in the literature. HCl-activated red mud is found to have an adsorption capacity of 6.207 mg/g at pH 4 and other optimum conditions of extraction [19]. Hydrogen peroxide-activated red mud is found to have an adsorption capacity of 64.79 mg/g at pH 6 [20]. Heat-activated red mud at 700°C is found to have an adsorption capacity of 38.2 mg/g at pH 4 [21]. Red mud coagulant is used to remove lead ions at pH 7, and its sorption ability is reported as 98.695 mg/g [22]. Zirconium-treated fine red mud impregnated in Zn-alginate beads (ZRMAB) is investigated for their adsorption nature towards phosphate from water, and its sorption ability for phosphates is found to be 13.64 mg/g of the adsorbent [23]. Organically modified magnesium silicate nanocomposites are also used for the removal of lead ions from water [24].

In this investigation, an adsorbent is prepared by doping hydrazine sulphate-treated red mud in calcium alginate beads, and its adsorption nature towards lead ions is studied. The present developed adsorbent is found to have 138.6 mg/g adsorption capacity towards Pb^2+^ ions, and further, the entrapping of the adsorbent in the beads facilitates the easy filtration.

## 2. Materials and Methods

### 2.1. Chemicals

The AR grade chemicals, namely, lead nitrate, sodium alginate, calcium chloride, nitric acid, hydrochloric acid, and sodium hydroxide, were purchased from SD Fine Chemicals Pvt. Ltd. and Merck & Co. Double distilled water was used for preparation of solutions.

### 2.2. Adsorbent

The red mud was obtained from Vedanta Aluminium Ltd., Utkal Alumina, Lanjigarh Refinery, Rayagada, Orissa, and the chemical composition of the raw red mud was presented in Table 1.

### 2.3. Treatment

The collected raw red mud sample was repeatedly washed with distilled water till the washings were neutral to pH and dried for two hours at 378 K. Then, the red mud was grounded and sieved to 75 *µ*. Thus obtained fine red mud was mixed with 1% hydrazine sulphate solution in 1 : 2 (w/w) ratio, and the resulting solution was boiled for 2 hrs. Then, the red mud was filtered, repeatedly washed with double distilled water, dried in hot-air oven at 378 K for two hours, and then stored in airtight coloured bottle for further work.

### 2.4. Immobilization of Treated Fine Red Mud in Calcium Alginate Beads

**Figure 1:**
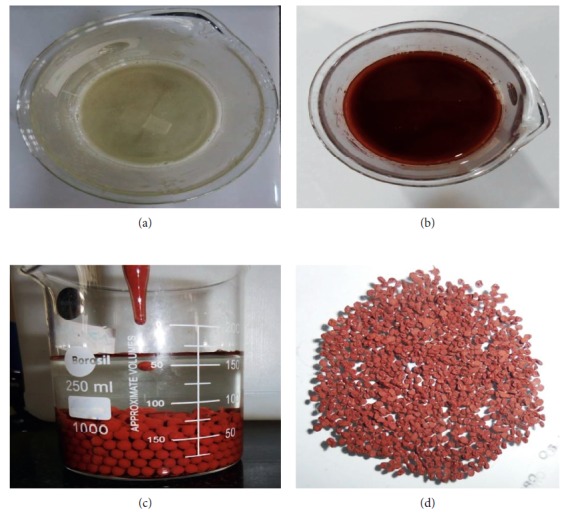
Hydrazine sulphate-treated calcium alginate beads. (a) 3.0% sodium alginate solution. (b) Treated red mud and sodium alginate mixture. (c) Formation of Ca-alginate beads doped with treated red mud. (d) Dried adsorbent (HRMCAB) at 343 K used for investigation.

A 100 mL of double distilled water was taken in a 250 mL beaker and to it, 3.0 g of sodium alginate powder was slowly added by continuous stirring at 363 K temperature and maintaining 1000 rpm until the solution was homogeneous and clear. Then, 0.1 g of hydrazine sulphate-treated red mud was slowly added to it by constant stirring, and the resulting mixture was cooled to room temperature. Then, this solution was dropwisely added to a supercooled (−2°C) 2% calcium chloride solution by maintaining uniform size of beads. The beads thus formed were filtered, repeatedly washed with distilled water for removing any remaining CaCl_2_ on beads, dried at 343 K, and stored in a coloured bottle (Figure 1).

### 2.5. Surface Characterization

**Figure 2:**
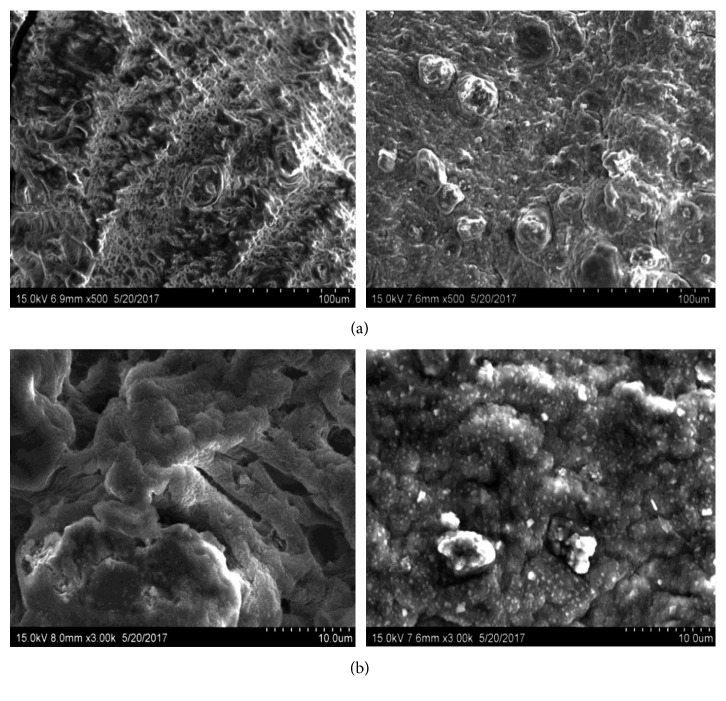
SEM images of adsorbent (HRMCAB). (a) SEM images of adsorbent before (left) and after (right) lead adsorption at 500x resolution. (b) SEM images of adsorbent before (left) and after (after) lead adsorption at 3000x resolution.

FESEM images of the adsorbent (HRMCAB) were taken by using HITACHI S-3700N SEM instrument manufactured by HITACHI High-Technologies Ltd., India. The magnifications from 500 to 15,000x and accelerating voltage of 15,000 V were maintained while taking the SEM images, and the images are presented in Figure 2.

**Figure 3:**
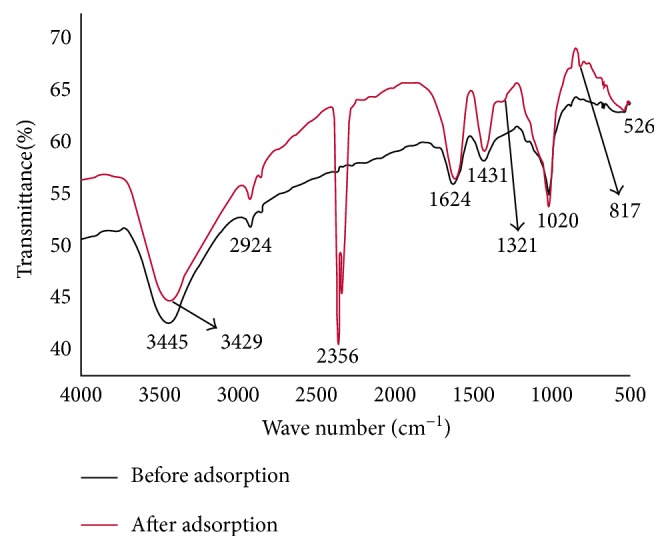
FTIR spectra of adsorbent (HRMCAB).

FTIR spectra of HRMCAB (before and after adsorption) were recorded using Shimadzu (8400S) FTIR Spectrophotometer. The spectrum was recorded by adopting the KBr pellet method in the range 4000 to 500 cm^−1^ at room temperature and at optical resolution of 4 (1/cm). The observed spectrum is presented in Figure 3.

**Figure 4:**
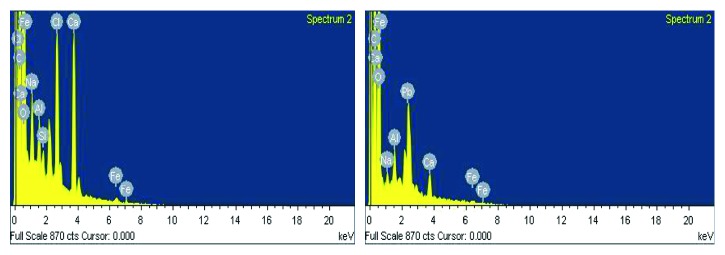
EDX spectra of adsorbent (HRMCAB) before and after adsorption of lead.

EDX spectrum of the adsorbent before and after adsorption of lead was recorded by using Hitachi (S-3700N) EDX detector and is presented in Figure 4.

**Figure 5:**
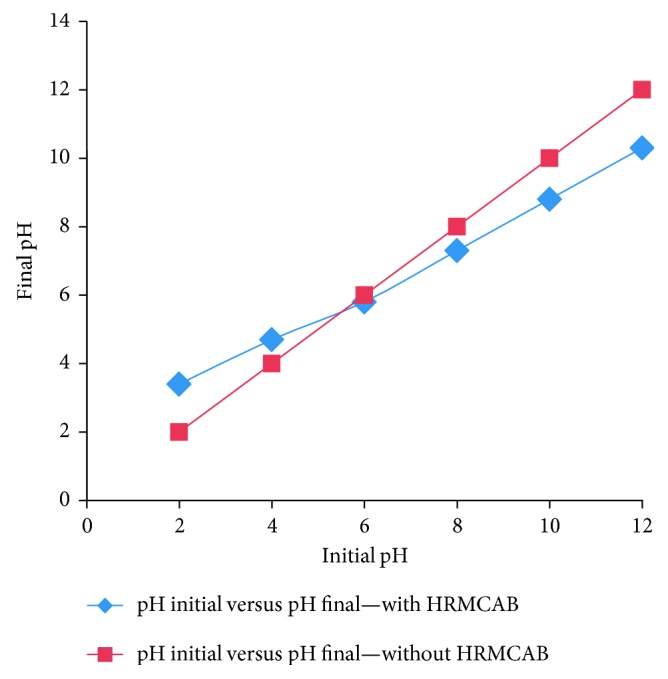
pH_zpc_ of the adsorbent.

By adopting the pH equilibrium method [25, 26], pH_ZPC_ of the adsorbent (HRMCAB) was determined by using Hanna pH meter, model HI2211-02, and the obtained plot is presented in Figure 5.

### 2.6. Method

Batch modes of extractions were adopted [27–29]. 100 mL of 100 mg/L lead solution was taken in 250 mL conical flasks and to it, different quantities of adsorbent (0.5 to 3 g) were added. Then, using 0.1 M HCl and 0.1 M NaOH solutions, the pHs were adjusted to 2 to 12, and the conical flasks were shaken using the orbital shaker at 300 rpm for 240 min at 303 K. After the completion of the required time, the conical flasks were removed, and their contents were filtered. The remaining Pb^2+^ ions in the solution were analysed by atomic adsorption spectroscopy (AA 500) at different parameters such as measure method: flame absorption, wavelength: 217.00 nm, slit: 0.4 nm, high voltage: 416.25 V, lamp current: 5.0 mA, and fuel flow rate: 1200 mL/min.

The percentage removal of lead and adsorbed amount of lead was calculated by using the following equations.

Adsorbed amount:

(1) $$\begin{array}{c} \left(q_{e}\right)=\frac{\left(C_{i}-C_{e}\right)}{m}V, \end{array}$$

percentage removal:

(2) $$\begin{array}{c} \left(\%R\right)=\frac{\left(C_{i}-C_{e}\right)}{C_{i}}100, \end{array}$$

where *C*_*i*_ is the initial Pb^2+^ concentration (mg/L), *C*_*e*_ is the Pb^2+^ concentration at equilibrium (mg/L), *V* is the volume of Pb^2+^ solution (simulated) in litres, and *m* is the mass of the adsorbent in grams.

The same procedure was adopted in finding the effect of the various physicochemical parameters on the extraction of Pb^2+^ “onto” the surface of HRMCAB. The results are presented in Figures 1–9 and Tables 2–4.

## 3. Results and Discussion

The raw red mud composition is presented in Table 1. It mainly consists of alumina, iron oxide, SiO_2,_ Na_2_O, CaO, and traces of P and V. The red mud is treated with hydrazine sulphate and is doped in calcium alginate beads. Thus obtained beads (HRMCAB) are characterized and studied for its adsorption nature towards lead ions from water. The results are presented below.

### 3.1. HRMCAB Characterization

#### 3.1.1. FESEM

FESEM images of the adsorbent (HRMCAB) before and after equilibration are noted and presented in Figure 2. It can be observed in SEM images that the adsorbent surface have many pores, edges, cavities, and corners before equilibration. Further, some crystalline aggregates are present, indicating different mineral phases such as gibbsite, goethite, and hematite.

After equilibration of the adsorbent with waters containing lead ions, there is an emphatic change on the SEM photographs. The pores and cavities are clogged, edges disappeared, and phase boundaries are blurred. Further, some shiny patches appeared. All these changes in the surface features of the adsorbent indicate that the lead is “onto” the surface of the adsorbent.

**Figure 6:**
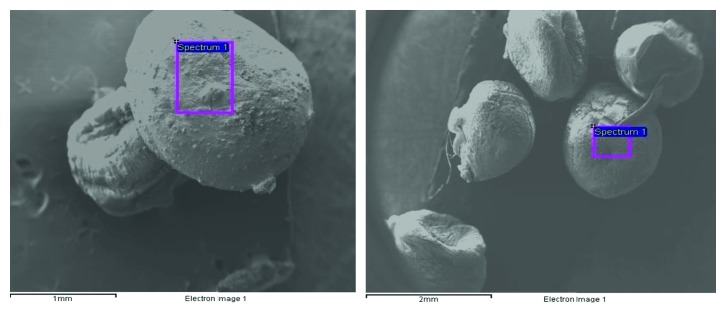
Electronic images of the adsorbent (HRMCAB) before and after lead adsorption.

Moreover, the electronic images presented in Figure 6 also indicate that the lead is present on the adsorbent surface.

#### 3.1.2. FTIR

The FTIR spectrum of the adsorbent (before and after adsorption of the lead) is presented in Figure 3.

The band pertaining to the stretching –OH group of silanol groups and also adsorbed water appeared at 3445 cm^−1^ as broad band in the before adsorption spectrum. In the after adsorption spectrum, this band is shifted to 3429 cm^−1^. The bending vibrations of –OH are assigned to the peak at 1624 cm^−1^ in both before and after adsorption spectra, and there is no change in their position. The peaks at 1020 cm^−1^ and 526 cm^−1^ in both spectra can be assigned to Al–O–Si (symmetric), Si–O–Si (asymmetric), and Fe–O stretching vibrations. The drastic difference in the spectral peaks before and after adsorption of lead could be seen in the appearance of an intensive band at 2356 cm^−1^ and small peaks at 1321 cm^−1^ and 817 cm^−1^ in the after adsorption spectrum. These features indicate that the lead is “onto” the surface of the adsorbent (HRMCAB).

#### 3.1.3. EDX Spectrum

The EDX spectra of adsorbent (HRMCAB) before and after adsorption of lead are presented in Figure 4. The presence of lead peak in the spectrum taken after adsorption equilibration and its absence before adsorption indicate that the lead ions are successfully adsorbed “onto” the surface of the adsorbent (HRMCAB).

### 3.2. Influence of Various Physicochemical Parameters

The sorption ability of the adsorbent (HRMCAB) towards Pb^2+^ is investigated using simulated waters containing Pb^2+^ and varying different physicochemical conditions, namely, pH, equilibration time, adsorbent dosage, initial Pb^2+^ concentration, temperature, and interfering co-ions. The results obtained are presented below.

#### 3.2.1. Effect of pH

**Figure 7:**
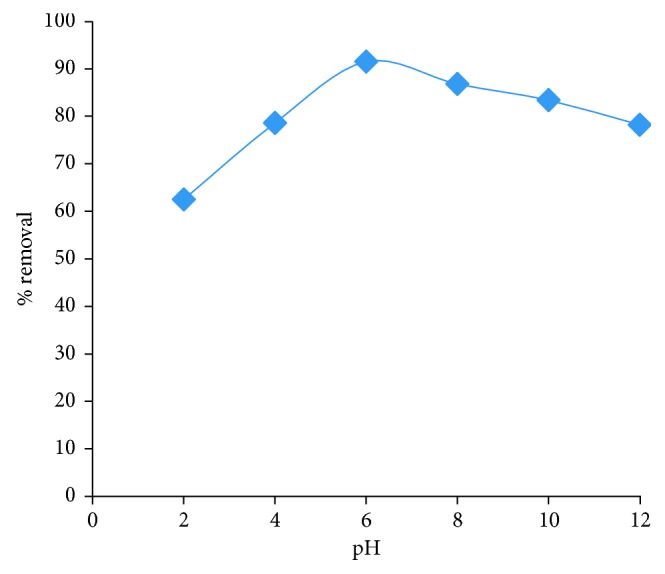
Effect of pH on adsorption of lead.

Behaviour of the adsorbent (HRMCAB) towards lead ions mainly depends upon the solution pH. Hence, by varying the pH from 2.0 to 12.0, the optimum pH for the successful removal of lead is investigated while keeping the other conditions of extraction at other ideal levels, namely, sorbent dosage: 20 g/L, agitation time: 180 min, rpm: 300, initial concentration: 100 mg/L, and temperature: 303 K. The results are presented in Figure 7.

It can be seen from Figure 7 that the maximum removal of Pb^2+^ to an extent of 91.5% can be removed at pH 6. But, as the pH is increased to 8, 10, and 12, the % removal decreased to 86.8%, 83.4%, and 78.2%, respectively. But, as the pH is decreased to 4 and 2, the % removal is decreased to 78.6% and 62.5%, respectively. It can be seen that on the acidic side of the pH, the fall of % removal is more pronounced than on the basic side. These observations can be accounted from the view point of pH_ZPC_ and the speciation of Pb^2+^ ion at various pHs. From Figure 5, it may be inferred that pH_ZPC_ is 5.8, and hence, at this pH, no ionic thrust prevails on HRMCAB surface. Below this pH, protonation to surface hydroxyl groups of HRMCAB occurs, thereby imparting +ve charge to the surface. The lead species in acidic conditions is Pb^2+^, and as it is a cation, it is to compete with H^+^ ions for being adsorbed onto the surface of the adsorbent. As H^+^ ions are more facile, the Pb^2+^ is less adsorbed, and hence, the % removal is less. On other hand, as the pH is above 6, the surface of the adsorbent acquires −ve charge. But the lead at pH above 8 is in the form of an anion PbO_2_^2−^. Hence, the negative surface charge will repel PbO_2_^2−^, and so, the % removal is decreased. The maximum % removal is found at pH 6 when neutral conditions prevail on the surface of the adsorbent and lead is in cationic speciation.

#### 3.2.2. Contact Time

**Figure 8:**
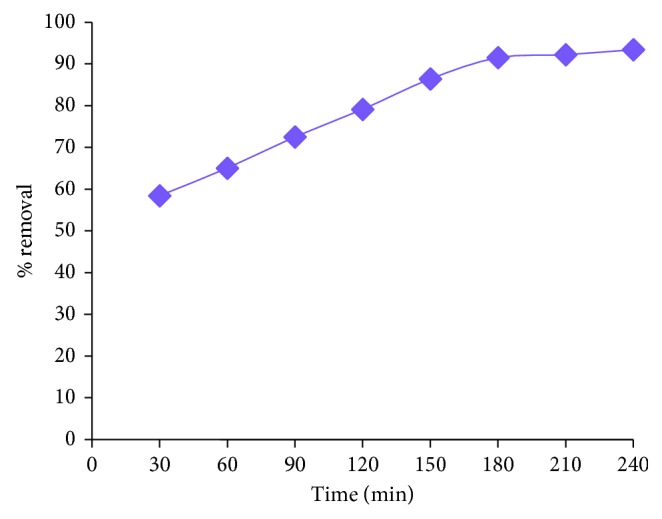
Effect of contact time on adsorption of lead.

The % removal of lead at different time intervals is shown in Figure 8. As seen from the figure, the extraction of lead increases from 58.4% to 91.5% until it reaches the steady state value 180 min with increase in time. After reaching the equilibrium state, the % removal remains almost constant even when the agitation time is increased to 240 min. Initially, rapid adsorption is occurred due to availability of more vacant sites. But with increase in time, the vacant sites are used up, and hence, the removal is slowed down.

#### 3.2.3. Adsorbent Dosage

**Figure 9:**
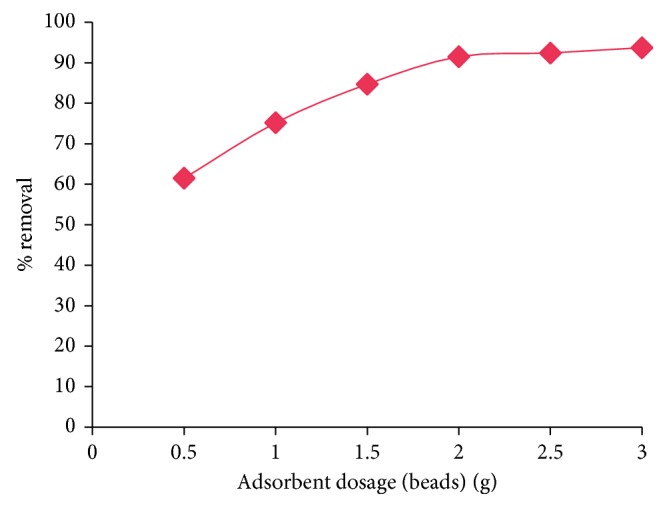
Effect of sorbent dosage on adsorption of lead.

The variation of % removal with increase in the adsorbent dosage is investigated when all other extraction conditions are maintained at optimum levels. The results are presented in Figure 9. It can be noted that as the dosage increased from 0.5 g to 2.0 g of beads/100 mL, the % removal is increased from 61.5% to 91.5%, but after that, the % removal remains constant. The optimum dosage is 2.0 g/100 mL (in terms of beads).

#### 3.2.4. Initial Pb^2+^ Concentration

**Figure 10:**
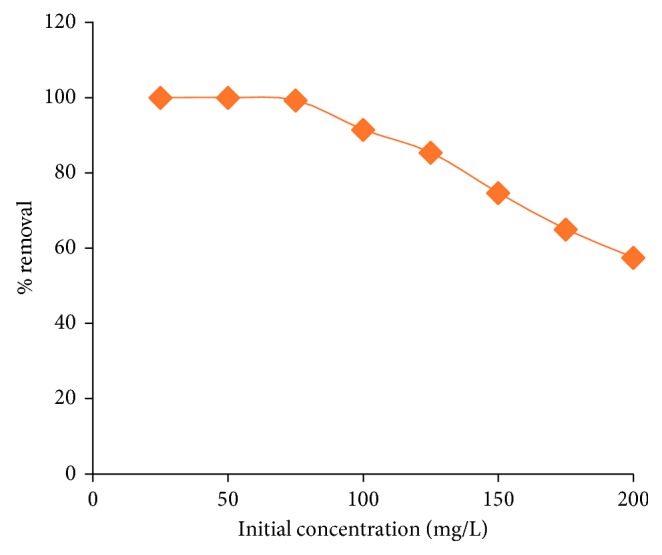
Effect of initial concentration on adsorption of lead.

By changing the initial concentration of Pb^2+^ from 25 mg/L to 200 mg/L, but keeping all other extraction conditions at their optimum levels, the influence of Pb^2+^ initial concentration on the extraction of Pb^2+^ is investigated. The results are presented in Figure 10. It can be inferred from the figure that as the Pb^2+^ concentration increases from 25 mg/L to 200 mg/L, the % removal decreases from 100.0% to 57.0%.

At lower concentrations of lead ions, the availability of binding sites of the adsorbent (HRMCAB) is more, and hence, extraction of lead ions is more. With the fixed amount of adsorbent, only a definite amount of adsorption sites is available, and hence, as the initial concentration of lead ion increases, there is a multiple competition to secure the sorption sites, and this results in the decrease in the % removal.

#### 3.2.5. Co-Ions

**Figure 11:**
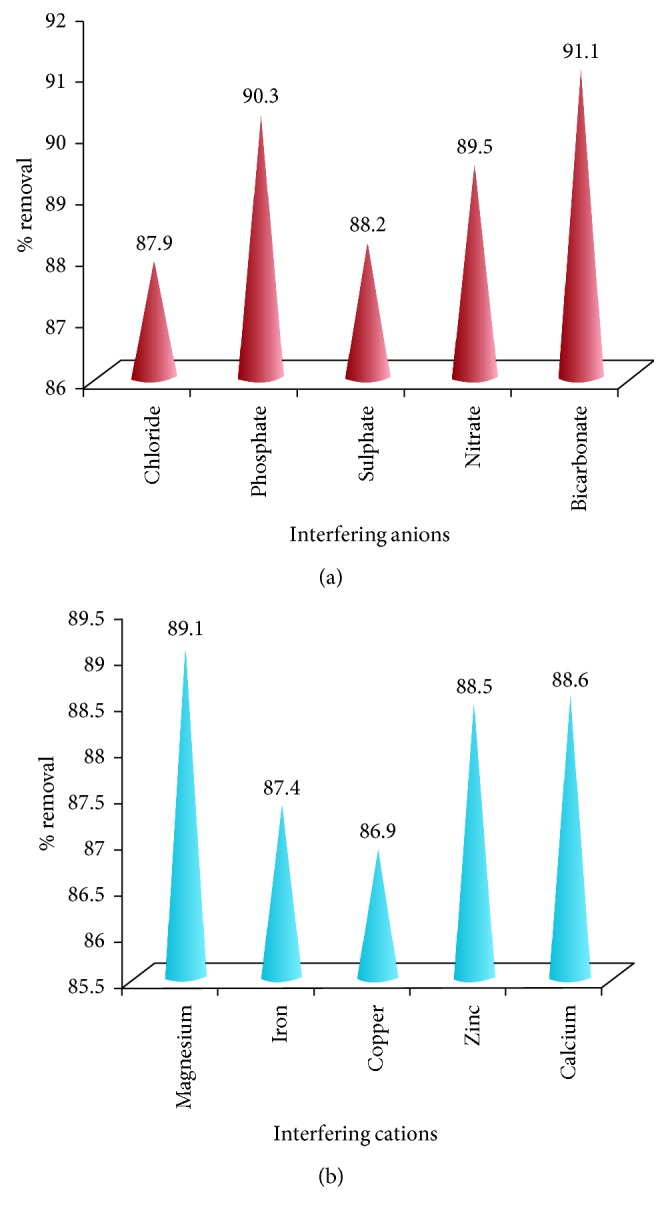
(a) Effect of interfering anions on adsorption of lead. (b) Effect of interfering cations on adsorption of lead.

The interference of commonly found co-ions (fivefold excess) in water on adsorption of the Pb^2+^ is studied, and the results are depicted in Figures 11 and 11.

It can be seen in the case of co-cations that the interference is of the order Cu^2+^ > Fe^3+^ > Zn^2+^ > Ca^2+^ > Mg^2+^. The interference in the case of anions is in the order Cl^−^ > SO_4_^2−^ > NO_3_^−^ > PO_4_^3−^ > HCO_3_^−^.

#### 3.2.6. Thermodynamic Parameters

**Figure 12:**
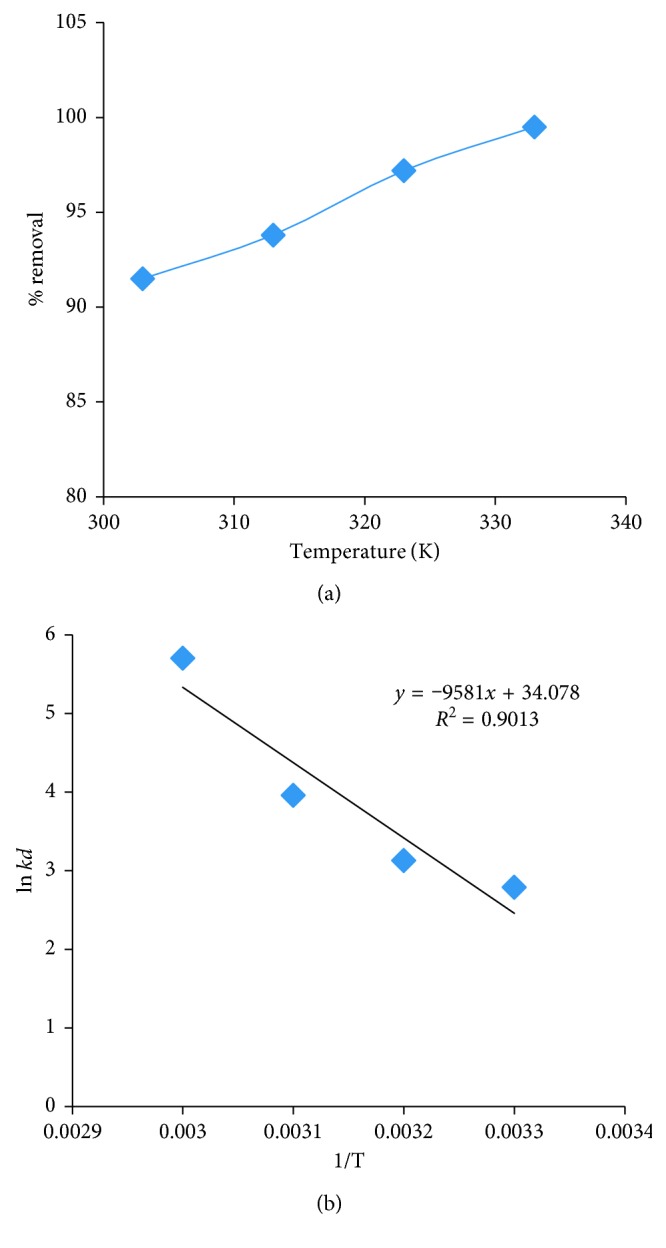
(a) Effect of interfering temperature on adsorption of lead. (b) Effect of interfering cations on adsorption of lead.

The effect of temperature on the % of extraction of Pb^2+^ is studied by increasing the temperature in the intervals of 10 K in the range 303 to 333 K, while keeping all other conditions of extraction at optimum levels, namely, pH: 6, contact time: 180 min, initial concentration of lead: 100 mg/L, rpm: 300, and adsorbent dosage: 20 g/L. The obtained results are presented in Figures 12 and 12 and Table 2. The % of extraction is found to be increased from 91.5% to 99.5% as the temperature increases from 303 to 333 K.

Thermodynamic parameters, namely, enthalpy change (∆*H*) (kJ/mole), entropy change (∆*S*), and free energy change (∆*G*) (kJ/mole), are evaluated at different temperatures using the equations Δ*G*^0^=Δ*H*^0^ − *T*Δ*S*^0^;*K*_*d*_=*q*_*e*_/*C*_*e*_, Δ*G*^0^=−*RT* ln *K*_*d*_;ln *K*_*d*_=Δ*S*^0^/*R* − Δ*H*^0^/*RT*, where *C*_*e*_ is the equilibrium concentration of lead ion solution, *q*_*e*_ is the amount of adsorbed adsorbate Pb^2+^, *K*_*d*_ is the distribution coefficient of the adsorption, *R* is the gas constant, and *T* is the absolute temperature in Kelvin as described in the literature [30, 31]. The values obtained are presented in Table 2.

The positive Δ*H* value (79.656) indicates the endothermic and physisorption nature of the adsorption process. The negative Δ*G* values reflect that the nature of the adsorption is spontaneous. Moreover, the “+” value of Δ*S* reflects the increase in randomness at the boundary of solid and liquid during the equilibration leading to more adsorption of lead ions.

### 3.3. Adsorption Isotherms

Freundlich [32], Langmuir [33], Temkin [34], and Dubinin–Radushkevich [35] models related to adsorption equilibrium are investigated as described in the literature to understand the nature and mode of adsorption.

**Figure 13:**
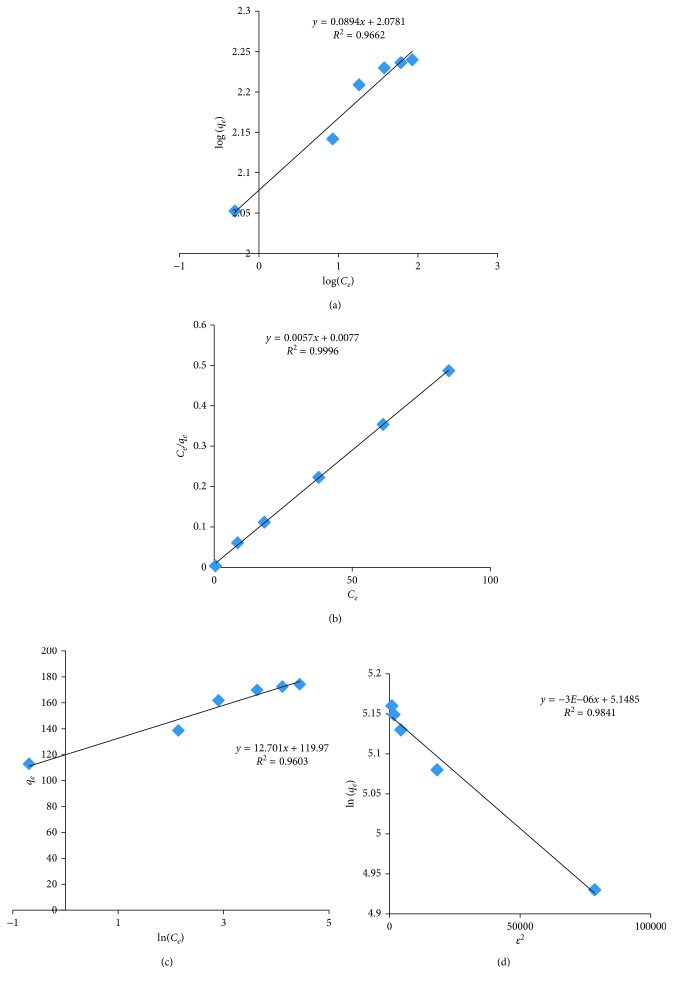
(a) Freundlich adsorption isotherm. (b) Langmuir adsorption isotherm. (c) Temkin adsorption isotherm. (d) Dubinin–Radushkevich adsorption isotherm.

The linear equations used for Freundlich model is log(*q*_*e*_)=log *k*_*f*_+(1/*n*)log *C*_*e*_ and for Langmuir model is (*C*_*e*_/*q*_*e*_)=(*a*_*L*_/*k*_*L*_)*C*_*e*_+1/*kL*, where *C*_*e*_, *n*, and *q*_*e*_ are adsorption capacity (mg/g), empirical parameter, and amount of Pb^2+^ adsorbed, respectively, and *k*_*L*_ and *a*_*L*_ are the Langmuir constants. The obtained results are presented in Figures 13 and 13 and Table 3. The dimensionless separation factor (*R*_*L*_) is calculated using the equation *R*_*L*_=1/(1+*a*_*L*_*C*_*i*_) [36]. *R*_*L*_ values indicate the nature of adsorption: *R*_*L*_ = 1, linear; *R*_*L*_ > 1, unfavourable; *R*_*L*_ = 0, irreversible; and 0 < *R*_*L*_ < 1, favourable. The *R*_*L*_ values of the present equilibration system are found to be between 0 and 1 (Table 3), and this indicated that lead ions are favourably adsorbed onto the surface of HRMCAB.

The *R*^2^ values of Freundlich model and Langmuir model are 0.9662 and 0.9996, respectively. This implies that Langmuir adsorption isotherm model is more acceptable, indicating the homogeneous nature of the adsorbent and the monolayer formation of lead onto the HRMCAB surface.

Further, Temkin and Dubinin–Radushkevich isotherms are also used in analysing the equilibrium process. Temkin equations used are *q*_*e*_=*B* ln *C*_*e*_+*B* ln *A*and*B*=*RT*/*b*, where *A*  is the Temkin isotherm constant (L/g), *B* is the heat of sorption (J/mol), *b* is the Temkin isotherm constant, *T* is the temperature (*k*), and *R* is the gas constant.

Linear form of Dubinin–Radushkevich equation is ln *q*_*e*_=−*βε*^2^+ln *q*_*m*_, where *ε*=*RT* ln (1+1/*C*_*e*_), *β* is the energy constant, and *q*_*m*_ is the Dubinin–Radushkevich monolayer adsorption capacity (mol/g). The linear plots of these two isotherms are presented in Figures 13 and 13, and the correlation coefficient and isothermal constants are presented in Table 3. By using the formula *E* = 1/$\sqrt{2}\beta$ and by the slope of the Temkin isotherm constants, the heat of sorption (*B*) and mean free energy (*E*) are calculated. As *E* is <8 kJ/mol (i.e., 0.408) and *B* is <20 kJ/mol (i.e., 12.701), the mechanism of adsorption is “physisorption” in nature, that is, nonspecific adsorption attributed to the weak Van der Waals forces between HRMCAB and lead ions.

### 3.4. Kinetics of Adsorption

**Figure 14:**
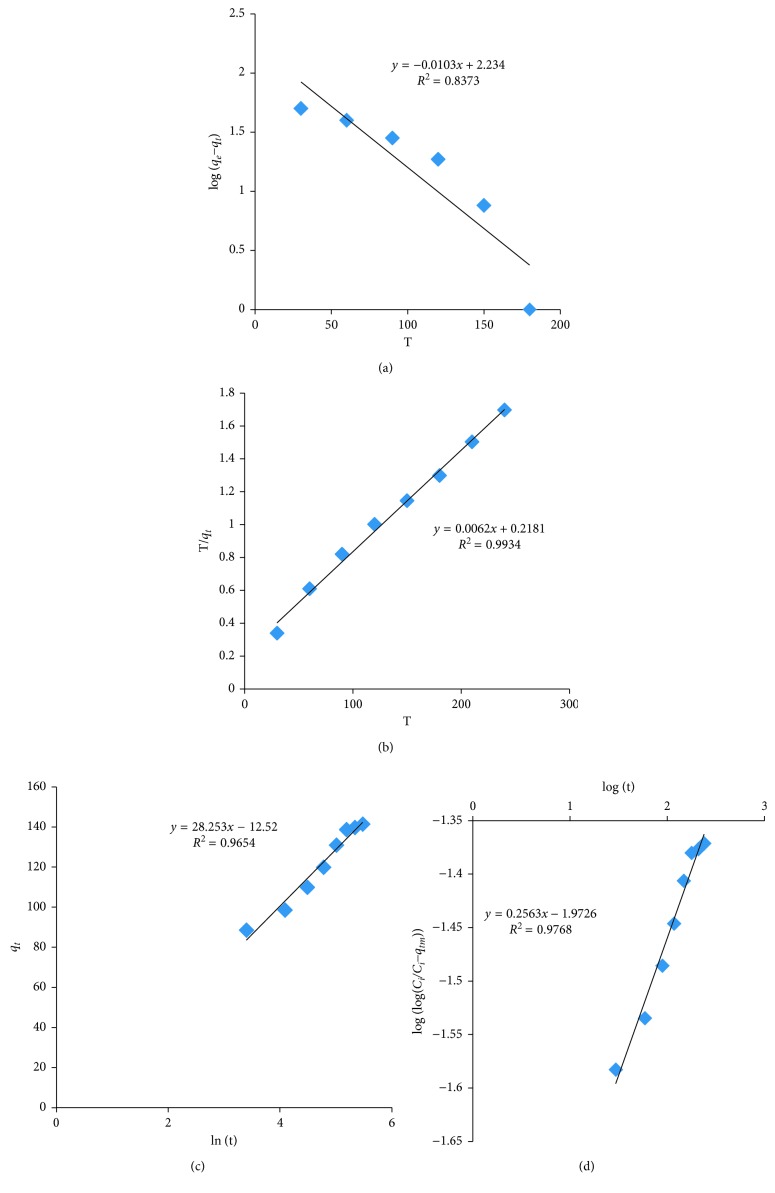
(a) Pseudo-first-order kinetics. (b) Pseudo-second-order kinetics. (c) Elovich model. (d) Bangham's pore diffusion model.

The adsorption kinetics are analysed by using pseudo-first order: log(*q*_*e*_ − *q*_*t*_)=log *q*_*e*_ − *K*_1_/2.303*t* [37], pseudo-second order: *t*/*q*_*t*_=1/*K*_2_*q*_*e*_^2^+1/*q*_*e*_*t* [38], Bangham's pore diffusion model: log[log(*C*_*i*_/*C*_*i*_ − *q*_*t*_*m*)]=log(*K*_*o*_/2.303*V*)+∝log(*t*) [39], and Elovich equation: *q*_*t*_=1/*β*ln(∝*β*)+1/*β*ln(*t*) [40]. The results are presented in Figures 14–14 and Table 4. The correlation coefficient value (*R*^2^) is found to be in the following order: pseudo-second order (0.9934) > Bangham's pore diffusion model (0.9768) > Elovich model (0.9654) > pseudo-first order (0.8373). Hence, the pseudo-second-order model is better fit to explain the adsorption process.

### 3.5. Applications

The methodology developed in the present work is applied to the effluent samples collected at lead-based industries. The results are presented in Table 5. It can be inferred from the table that substantial amounts of lead can be removed from the industrial samples.

### 3.6. Comparison

The adsorption ability of the adsorbent developed in this work is compared against various hitherto reported adsorbents in the literature as presented in Table 6. It may be inferred that the present developed adsorbent has good adsorption ability than many adsorbents developed so far, and further, the immobilization of the hydrazine sulphate-treated red mud in calcium alginate beads renders the filtration easy.

### 3.7. Regeneration and Reuse

Of the various leaching agents tried for the extraction of lead from the adsorbent, 0.1 M HCl is found to be good and hence adopted in this work.

**Figure 15:**
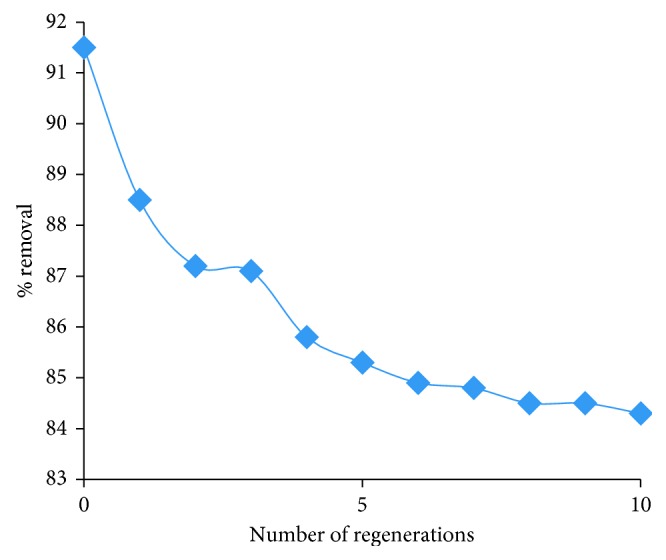
Number of regenerations versus % removal.

A number of regenerations of the adsorbent are made, and thus regenerated adsorbents are used for the removal of lead. The results obtained are presented in Figure 15. It is seen that, as the number of regenerations increases, the adsorption nature decreases. But even with 10th regenerated adsorbent, Pb^2+^ is successfully removed to an extent of 84.3%. Hence, by repetitive use of the regenerated adsorbent, the lead can be completely removed from water samples at the comfortable natural water pH 6. This indicates that the present developed methodology is successful.

## 4. Conclusions

Hydrazine sulphate-activated red mud doped in calcium alginate beads (HRMCAB) are prepared and are used as sorbent to extract lead ions from water by optimising various physicochemical parameters. Batch methods of extractions are adopted. 91.5% of lead is found to be removed from simulated water at pH 6, sorbent dosage of 0.066 g of red mud (doped in 2.0 g of HRMCAB beads)/100 mL, agitation time of 180 min, initial concentration of 100 mg/L, 300 rpm, and 303 K temperature. The adsorption capacity of HRMCAB is 138.63 mg/g. The interference of the co-cations is of the order Cu^2+^ > Fe^3+^ > Zn^2+^ > Ca^2+^ > Mg^2+^ while the anions interfered in the order Cl^−^ > SO_4_^2−^ > NO_3_^−^ > PO_4_^3−^ > HCO_3_^−^.

Surface morphological studies adopting FTIR, FESEM, and EDX confirm that lead is onto the surface of the adsorbent. By evaluating thermodynamic parameters, it is inferred that the sorption process is spontaneous and “physisorption” in nature.

On analysis of the various adsorption isotherms, the adsorption follows the Langamuir isotherm model, thereby confirming the homogeneous surface of the adsorbent and monolayer formation. Further, adsorption kinetics is analysed adopting various models, and it is observed that it follows pseudo-second order with *R*^2^ = 0.9934.

By immobilizing the activated red mud in the beads, the filtration process is made easy. Successive regeneration of the adsorbent with 0.1 M HCl has not markedly affected the adsorption nature of the adsorbent. Even after 10 cycles of regeneration of the adsorbent, substantial amount of lead is removed to an extent of 83.4%. Hence, by repetitive use of the same adsorbent, it is possible to remove lead completely from the water. Further, the immobilization of activated red mud in the beads facilitated the easy filtration. The methodology developed is successfully applied for removing lead from industrial waste waters.

## Figures and Tables

**Table 1:** Chemical properties of red mud.

Parameter	Result (%)
Alumina as Al_2_O_3_	15.47
Iron as Fe_2_O_3_	58.78
Silica as SiO_2_	6.58
Titanium as TiO_2_	4.39
Soda as Na_2_O	3.63
Calcium as CaO	1.49
Phosphorus as P_2_O_5_	0.159
Vanadium as V_2_O_5_	0.110
Loss of ignition (105–1000°C)	7.22

**Table 2:** Thermodynamic parameters of adsorption of the lead on HRMCAB.

*T*(*K*)	*K*_*d*_ (L/g)	Vant Hoff equation: ln (*K*_*d*_) = (Δ*S*^0^/*R*) − (Δ*H*^0^/*RT*)	Δ*H* (kJ/mole)	Δ*S* (J/mole)	*R* ^2^	Δ*G* (kJ/mole)
303	16.30	*y* = −9581*x* + 34.078	79.656	283.32	0.9013	−6.189
313	22.92					−9.022
323	52.59					−11.855
333	301.51					−14.689

**Table 3:** Adsorption parameters.

S. number	Adsorption isotherms	—	Slope	Intercept	*R* ^2^
(1)	Freundlich isotherm	—	0.0894	2.0781	0.9662
(2)	Langmuir isotherm	*R*_L_ = 0.0133	0.0057	0.0077	0.9996
(3)	Temkin isotherm	*B* = 12.701	12.701	119.97	0.9603
(4)	Dubinin–Radushkevich isotherm	*E* = 0.408	−3*E*−06	5.1485	0.9841

**Table 4:** Adsorption kinetic parameters.

S. number	Adsorption kinetic parameters	Slope	Intercept	*R* ^2^
(1)	Pseudo-first-order model	−0.0103	2.234	0.8373
(2)	Pseudo-second-order model	0.0062	0.2181	0.9934
(3)	Elovich model	28.253	−12.52	0.9654
(4)	Bangham's pore diffusion model	0.2563	−1.9726	0.9768

**Table 5:** Lead concentration (before and after) of samples (samples collected from the effluents of lead-based industries in Andhra Pradesh, India).

S. number	Water samples	*C*_*i*_ (mg/l) (initial concentration of lead ions)	*C*_*f*_ (mg/l) (final concentration of lead ions)	% removal
(1)	Sample 1	4.24	0.42	89.9%
(2)	Sample 2	3.62	0.27	92.4%
(3)	Sample 3	2.89	0.16	94.3%
(4)	Sample 4	2.06	0.06	96.7%

**Table 6:** Comparison of maximum adsorption capacity of the adsorbent with other adsorbents from the literature.

S. number	Adsorbent	pH	*q*_*e*_ (mg/g)	Reference
(1)	HRMCAB	6.0	138.63	Present work
(2)	HCl-activated red mud	4.0	6.207	Kumar Sahu et al. [19]
(3)	H_2_O_2_-treated red mud	4.0	64.79	Gupta et al. [20]
(4)	Red mud coagulant	7.0	98.695	Kong [21]
(5)	Fluted pumpkin seed shell active carbon	—	14.286	Okoye et al. [41]
(6)	Pine cone-activated carbon	6.7	27.53	Momčilović et al. [42]
(7)	Coconut shell carbon	—	30.0	Sekhar [43]
(8)	Zinc chloride-activated tamarind wood	6.5	43.85	Acharyaa et al. [44]
(9)	Poly(acrylic acid)/bentonite nanocomposite	—	93.0	Rafiei et al. [45]
(10)	Magnetic magnetite (Fe_3_O_4_) nanoparticle	5.0	53.11	Rajput et al. [46]
